# Blood biochemical variables, antioxidative status, and histological features of intestinal, gill, and liver tissues of African catfish (*Clarias gariepinus*) exposed to high salinity and high-temperature stress

**DOI:** 10.1007/s11356-022-19702-0

**Published:** 2022-03-25

**Authors:** Mahmoud A. O. Dawood, Ahmed E. Noreldin, Hani Sewilam

**Affiliations:** 1 The Center for Applied Research on the Environment and Sustainability, The American University in Cairo, Cairo, 11835 Egypt; 2 Animal Production Department, Faculty of Agriculture, Kafrelsheikh University, Kafr El-Sheikh, 33516 Egypt; 3 Histology and Cytology Department, Faculty of Veterinary Medicine, Damanhour University, Damanhour, 22511 Egypt; 4 Department of Engineering Hydrology, RWTH Aachen University, Aachen, Germany

**Keywords:** Aquaculture, Salinity, Heat stress, Catfish, Oxidative stress

## Abstract

African catfish is a freshwater species with a high ability to resist brackish water conditions, but heat stress may impair the health status of fish. Thus, the impact of varying levels of water salinity (0, 4, 8, and 12 ppt) was investigated on the growth performance, survival rate, and blood biochemistry of African catfish (average weight: 180.58 ± 2.8 g and average length: 38 ± 1.2 cm) for 4 weeks; then, fish were stressed with high temperature (32 °C) for 72 h. The growth performance and survival rate were markedly higher in fish reared in 0, 4, and 8 ppt than fish in 12 ppt (*p* < 0.05). Before heat stress, the superoxide dismutase (SOD), catalase (CAT), glutathione (GSH) activities, and malondialdehyde (MDA) levels were markedly increased in fish stressed with 12-ppt salinity (*p* < 0.05). After heat stress, all groups showed a marked increased SOD, CAT, GSH, and MDA levels than fish before heat stress in the same manner (*p* < 0.05). Furthermore, fish in the 12 ppt group showed severe intestinal, gill, and liver histological features. The levels of blood glucose and cortisol were markedly increased in fish exposed with 8 and 12 ppt than 0 ppt gradually either before or after heat stress (*p* < 0.05). The highest values of ALT, AST, urea, creatinine, and the lowest total protein, albumin, and globulin were observed in fish reared in 12 ppt. Significant salinity and heat stress interactions were seen on the ALT, AST, urea, creatinine, total protein, albumin, and globulin values (*p* < 0.05). The integrated multi-biomarker response (IBR) results showed marked differences among the groups and increased gradually before and after heat stress, with the highest IBR in 12 ppt. In conclusion, growing African catfish in high salinity (12 ppt) hampered the growth performance and health status while the heat stress improved the antioxidative status vis-a-vis increased lipid peroxidation along with higher stress-related markers in expressed both blood and tissue.

## Introduction

Climate change is one of the main challenges associated with various impacts on humanity, animals, and the ecosystem (Galappaththi et al., 2020). Extremely low and high temperatures resulting from the fluctuations in climate change disrupt the biological and physiological rhymes of living organisms (Esam et al., 2022; Falconer et al., 2020). An observed rising in the temperature is markedly hitting vast areas around the globe for long periods throughout the year (Stewart-Sinclair et al., 2020). Interestingly, it becomes difficult to separate between the year four seasons due to the collapse of weather temperature and unclear temperature limits. As one of the major food suppliers, the aquaculture industry is not far away from the impacts of climate change (Ahmed and Turchini, 2021; Dawood et al., 2021a). Most aquatic animals require optimal water temperature to have healthy physiological and productive performances (Dawood, 2021; Zhou et al., 2021). High temperature is involved in impairing the reproduction and hatching of finfish seeds (Cai et al., 2020b; Pountney et al., 2020). High temperatures in adult fish induce deformities in the erythrocytes, causing nuclear and cellular damage (Islam et al., 2020). Under these circumstances, the regulations of growth, immunity, antioxidative, and antistress hormones and genes can be disturbed, leading to irregular growth performance and resistance to infection (Cai et al., 2020a; Dawood et al., 2020; Shahjahan et al., 2018).

Due to the temperature changes, the water salinity increases, particularly in the brackish water areas and places suffering from a lack of freshwater (Durigon et al., 2020; Thomas et al., 2020). Along with the fluctuations in the temperature, these uncontrolled water characteristics result in several physiological and biological abnormalities (Hlordzi et al., 2020; Magouz et al., 2022). High salinity levels alter the osmoregulation capacity of fish, leading to irregular metabolic rates and disturbances in physiological and immunological status (Britz and Hecht, 1989). Consequently, fish suffer from weak growth performance and feed utilization, causing low productivity and substantial economic loss (Abass et al., 2016). In channel catfish (*Ictalurus punctatus*), a freshwater fish model, the interactive impacts of high temperature and water salinity resulted in fluctuations in the expression of growth hormone, osmoregulation, and homeostasis (Abass et al., 2016). Although that European seabass (*Dicentrarchus labrax*) is euryhaline fish species, high temperature (33 °C) combined with hypersalinity caused low adaptation ability through high mortality rates and oxidative stress (Islam et al., 2020). Since freshwater fish species are sensitive to water salinity changes (Nepal and Fabrizio, 2020), it is crucial to investigate the combined impacts of high temperature and salinity on the growth performances, physiological, immunological, and antioxidative responses.

African catfish (*Clarias gariepinus*) can perform adequately if the water temperature is around 25–28 °C (Andrews and Stickney, 1972; Ogunji and Awoke, 2017). However, high temperatures adversely impact oxygen availability in the water (Buentello et al., 2000). Hot temperature (32 °C) reduces the solubility of oxygen in the water and eventually leads to low metabolic and physiological function, thereby low growth and death (Dutta, 1994; Prokešová et al., 2015). Concurrently, this study aimed at evaluating the combined effects of salinity and high temperature on the serum biochemical traits, antioxidant, and stress-related markers of African catfish. Besides, the study evaluated the impacts of salinity and high temperature-induced oxidative stress on the intestine, gill, and liver histological features.

## Materials and methods

### Acclimatization of fish

One-hundred-twenty adult African catfish weighing 180.58 ± 2.8 g with an average length of 38 ± 1.2 cm were obtained from a private farm located in Kafr El-Sheikh city and gently transported to The Center for Applied Research on the Environment and Sustainability, The American University in Cairo, Cairo, Egypt. Fish were treated and handled by following the ethical guidelines approved by the ethical committee of the Faculty of Agriculture, Kafrelsheikh University, Egypt. Upon arrival, fish were kept in two 1000-L plastic tanks and kept for adaptation for 2 weeks. The tanks were supplied with continuous aeration, and the water was replaced with fresh dechlorinated water daily. During the adaptation period and throughout the trial, fish-fed pellets of 30% crude protein manufactured by Skretting (Bilbis, El Sharqia Governorate, Egypt) up to the satiation level twice daily (08:00 and 15:00).

### Experimental procedures

#### Exposure to salinity stress

After acclimatization, fish were distributed in twelve 100-L plastic tanks with ten fish in each tank. The experimental tanks were provided with continuous aeration, and half of the water was changed daily with dechlorinated water. Every three tanks were considered an experimental group where fish were reared in water with 0, 4, 8, and 12 ppt. The water salinity was raised gradually at 2 ppt daily until reaching the proposed salinity levels. The saline water was prepared daily by mixing dry sea saline with fresh water and kept in stock tanks. The water quality was checked daily and recorded to confirm that the proposed salinity levels were applied. The water was exchanged with temperature adjusted and appropriate saline water (0, 4, 8, and 12 ppt). When the proposed levels of salinity (0, 4, 8, and 12 ppt) were confirmed, all fish were kept under experimental conditions for 4 weeks. Feed intake was recorded to calculate the feed conversion ratio (FCR). The water quality was detected by Orion Star™ A329 Portable Multiparameter Meter (Thermo Scientific™, Waltham, MA, USA) for salinity, temperature, dissolved oxygen, and pH. Total ammonia (TAN) levels were measured calorimetrically using the APHA (1912) standard method. The dissolved oxygen, pH, and total ammonia levels were not meaningfully impacted by the effects of varying salinity levels before or after heat stress and recorded 6.21 ± 0.12 mg/L, 7.22 ± 0.18, and 0.03 ± 0.001 mg/L, respectively. The salinity levels were recorded 0.21 ± 0.02, 4.21 ± 0.11, 8.32 ± 0.23, and 12.32 ± 0.32, respectively. Water temperature was significantly higher in all groups after heat stress (32.36 ± 0.41 °C) than before heat stress (26.95 ± 0.11 °C).

#### Exposure to heat stress

Using electrical heaters, the remaining fish in each tank were stressed with heat stress (32 °C) for 72 h. Each tank was fixed with a heater, and the temperature was raised gradually at 2 °C per hour until reaching the proposed degree; then, fish were kept for 72 h under the experimental conditions. The water quality was checked regularly using the same procedure mentioned above.

### Collection of blood and tissue sample for biochemical analysis including antioxidant (SOD, CAT, GSH) and damage indicator (MDA) as well as tissue samples for histology

After 4 weeks, all fish were starved for 24 h then weighed and counted to calculate the growth performance, feed conversion ratio, and survival rate using the following formulae:

$$\mathrm{Weight}\ \mathrm{gain}\ \left(\%\right)=\left(\left(\mathrm{final}\ \mathrm{weight}\ \left(\mathrm{g}\right)-\mathrm{initial}\ \mathrm{weight}\ \left(\mathrm{g}\right)\right)/\mathrm{initial}\ \mathrm{weight}\ \left(\mathrm{g}\right)\right)\times 100$$

$$\mathrm{Specific}\ \mathrm{growth}\ \mathrm{rate}\ \left(\mathrm{SGR}\right)=100\times \left[\ln\ \mathrm{final}\ \mathrm{weight}\ \left(\mathrm{g}\right)\hbox{--} \ln\ \mathrm{initial}\ \mathrm{weight}\ \left(\mathrm{g}\right)\right]/\mathrm{days}$$

$$\mathrm{Feed}\;\mathrm{conversion}\;\mathrm{ratio}\;\left(\mathrm{FCR}\right)=\mathrm{feed}\;\mathrm{intake}\;\left(\mathrm g\right)/(\left(\mathrm{final}\;\mathrm{weight}\;\left(\mathrm g\right)-\mathrm{initial}\;\mathrm{weight}\;\left(\mathrm g\right)\right)$$

$$\mathrm{Survival}\ \left(\%\right)=100\times \mathrm{final}\ \mathrm{number}/\mathrm{initial}\ \mathrm{number}\ \mathrm{of}\ \mathrm{fish}$$

After salinity exposure and heat stress, all fish were anesthetized with tricaine methanesulfonate (MS-222; 25 mg/L), and the blood was collected from 3 fish per tank from the caudal vein using 3-mL non-heparinized syringes. The collected blood was kept clotting at 4 °C; then, serum was separated at 1107 g/15 min at 4 °C and kept at −20 °C for further analysis. The intestines, gills, and livers were dissected from the fish for preparing the homogenate and stocked at −20 °C. The homogenates of collected tissues were prepared by rinsing the tissues in ice-cold phosphate-buffered saline (PBS) (50 mM potassium phosphate, pH 7.5 1 mM EDTA). Tissues were homogenized in 10-fold PBS buffer (1-g tissue, 1:10 w:v) with glass homogenizer tubes (pellet pestle motor) and centrifuged at 7871 *g* for 5 min. The supernatant was collected and stored at 4 °C for further analysis.

### Analysis of both blood and tissue samples

Serum aspartate aminotransferase (AST), alanine aminotransferase (ALT), creatinine, and urea were detected by SPIN 800 Autoanalyzer using readymade chemicals (kits) supplied by Spinreact Co. Spain, following the manufacturer’s instructions. Serum total proteins and albumins were determined, according to Doumas et al. (1981) and Dumas and Biggs (1972). Globulin was calculated by the difference between serum total protein and albumins. Glucose and cortisol levels were determined using glucose and cortisol enzymatic PAP kits obtained from Bio-Merieux (France) (Trinder, 1969).

Superoxide dismutase (SOD), catalase (CAT), and glutathione (GSH) in intestine, gill, and liver homogenate samples were measured using commercial kits following the manufacturer’s (Biodiagnostics Co., Egypt) instructions. The malondialdehyde (MDA) concentration was detected by following Uchiyama and Mihara (1978) and expressed as nmol MDA/g.

Intestines, gills, and livers were removed and flushed with phosphate buffer saline (PBS; pH 7.4) and fixed in neutral-buffered formaldehyde for 48 h. The fixed specimens were processed by the conventional paraffin embedding technique, including the dehydration through ascending grades of ethanol, clearing in three changes of xylene, and melted paraffin ended by embedding in paraffin wax at 65 °C. Four-micrometer-thick sections were stained by hematoxylin and eosin (H and E), as Bancroft and Layton (2013) described. The tissue histopathology examination was carried out using a digital camera (Leica EC3, Leica, Germany) connected to a microscope (Leica DM500) and with software (Leica LAS EZ).

### Integrated biomarker response and statistical treatment of data

The integrated biomarker response (IBR) was assessed using the measured biomarkers of African catfish exposed to high salinity and temperature. The IBR was applied only for the biomarkers showing meaningful differences among the groups by following Beliaeff and Burgeot (2002) and Iturburu et al. (2018). Several IBR indices were calculated from the same data changing the order of the biomarkers and using the median of all the index values as the final index value (Devin et al., 2014).

Levene’s test examined variance homogeneity of data to confirm the normality and homogeneity. All data were analyzed using one-way analysis of variance (ANOVA) by the SPSS 22.0 software by Duncan’s test. Differences were considered significant at *p* < 0.05. When significant differences were detected, two-way ANOVA was used to determine the effects of water salinity, heat stress, and their interaction on the water quality, blood biochemistry, and IBR of African catfish.

## Results

### Growth behavior

The final weight, weight gain, SGR, and survival rate were markedly higher in African catfish reared in 0, 4, and 8 ppt than fish in 12 ppt (*p* < 0.05; Table 1). Nevertheless, fish reared in 12 ppt had higher FCR than fish in 0, 4, and 8 ppt (*p* < 0.05; Table 1).

**Table 1:** Growth performance of African catfish exposed with varying levels of salinity

	0 ppt	4 ppt	8 ppt	12 ppt
IBW (g)	181.40 ± 1.22	179.73 ± 1.34	180.55 ± 1.26	180.65 ± 1.42
FBW (g)	235.00 ± 0.30 a	233.70 ± 1.80 a	233.28 ± 3.23 a	207.86 ± 5.13 b
WG (%)	29.55 ± 0.17 a	30.03 ± 1.00 a	29.20 ± 1.79 a	15.06 ± 2.84 b
SGR (%/day)	0.86 ± 0.02 a	0.88 ± 0.04 a	0.85 ± 0.05 a	0.47 ± 0.1 b
FCR	1.25 ± 0.03 c	1.31 ± 0.10 c	1.46 ± 0.03 b	3.46 ± 0.67 a
Survival (%)	100.00 ± 0.00 a	99.17 ± 0.83 a	97.50 ± 1.44 a	87.50 ± 2.89 b

Means ± S.E. in the same column with different letters differ significantly (*p* < 0.05). *IBW* initial body weight, *FBW* final body weight, *WG* weight gain, *SGR* specific growth rate, *FCR* feed conversion ratio

### Histopathological assessment in intestines, gills, and livers

**Fig. 1:**
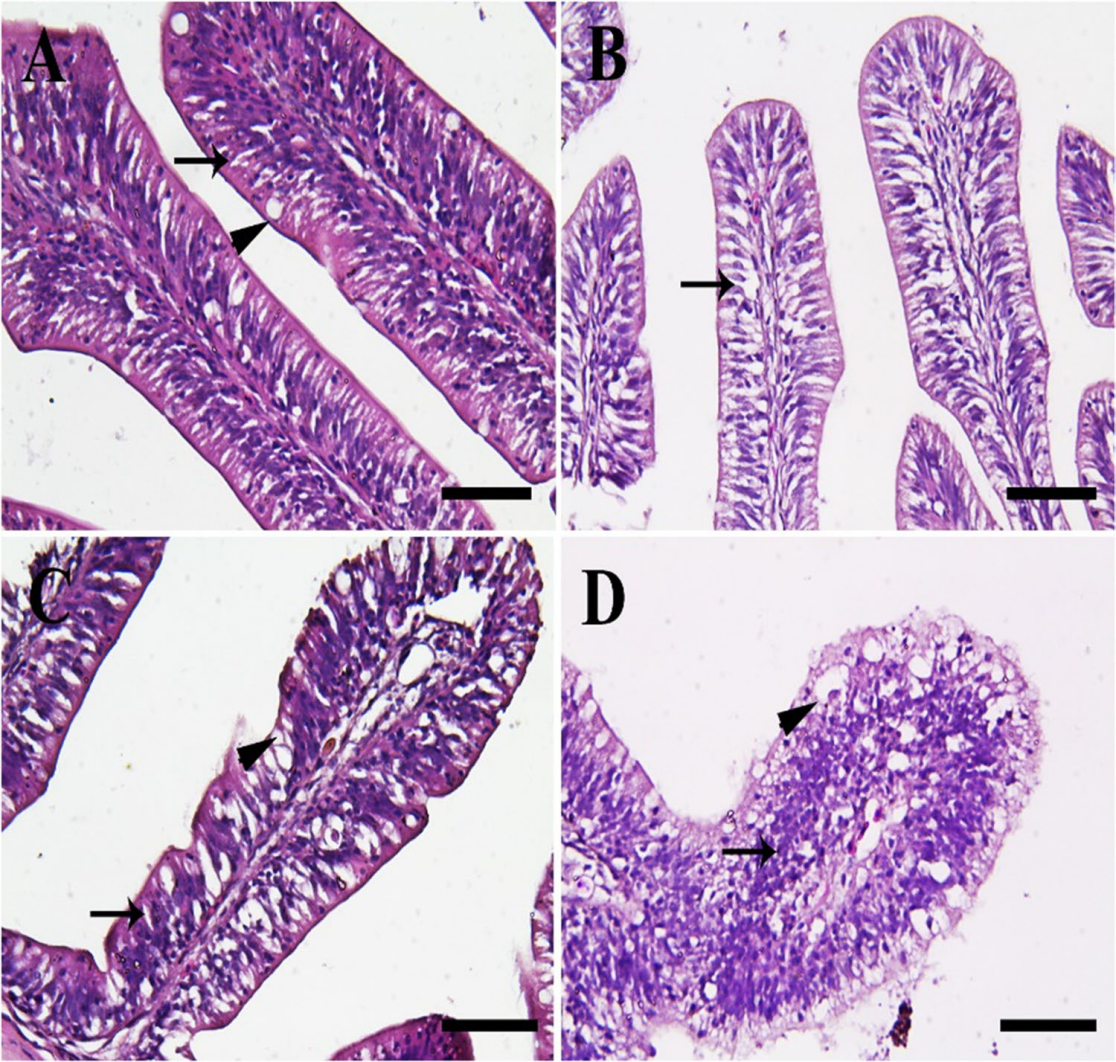
Histopathological examination of fish intestine. **A** The 0-ppt group revealing normal villi with normal enterocytes (thin arrow) and goblet cells (arrowhead). **B** Salinity (4 ppt) group revealing degenerative enterocytes (thin arrow). **C** Salinity (8 ppt) group showing necrosis in the enterocytes (thin arrow) and vacuolations (arrowhead). **D** Salinity (12 ppt) group exposing severe necrosis in the enterocytes with extensive vacuolations (arrowhead) and lymphocytic infiltrations (thin arrow). *Scale bar* = 50 μm

Fish reared in the 0-ppt group revealed normal intestinal architecture with normal villi (Fig. 1A). On the other hand, fish in the 4-ppt group showed slight degenerative changes in the enterocytes (Fig. 1B). Moreover, fish in the 8-ppt group exposed severe necrosis and vacuolations in the enterocytes (Fig. 1C). Furthermore, fish in the 12-ppt group revealed excessive necrosis and vacuolations in the enterocytes and massive lymphocytic infiltration (Fig. 1D).

**Fig. 2:**
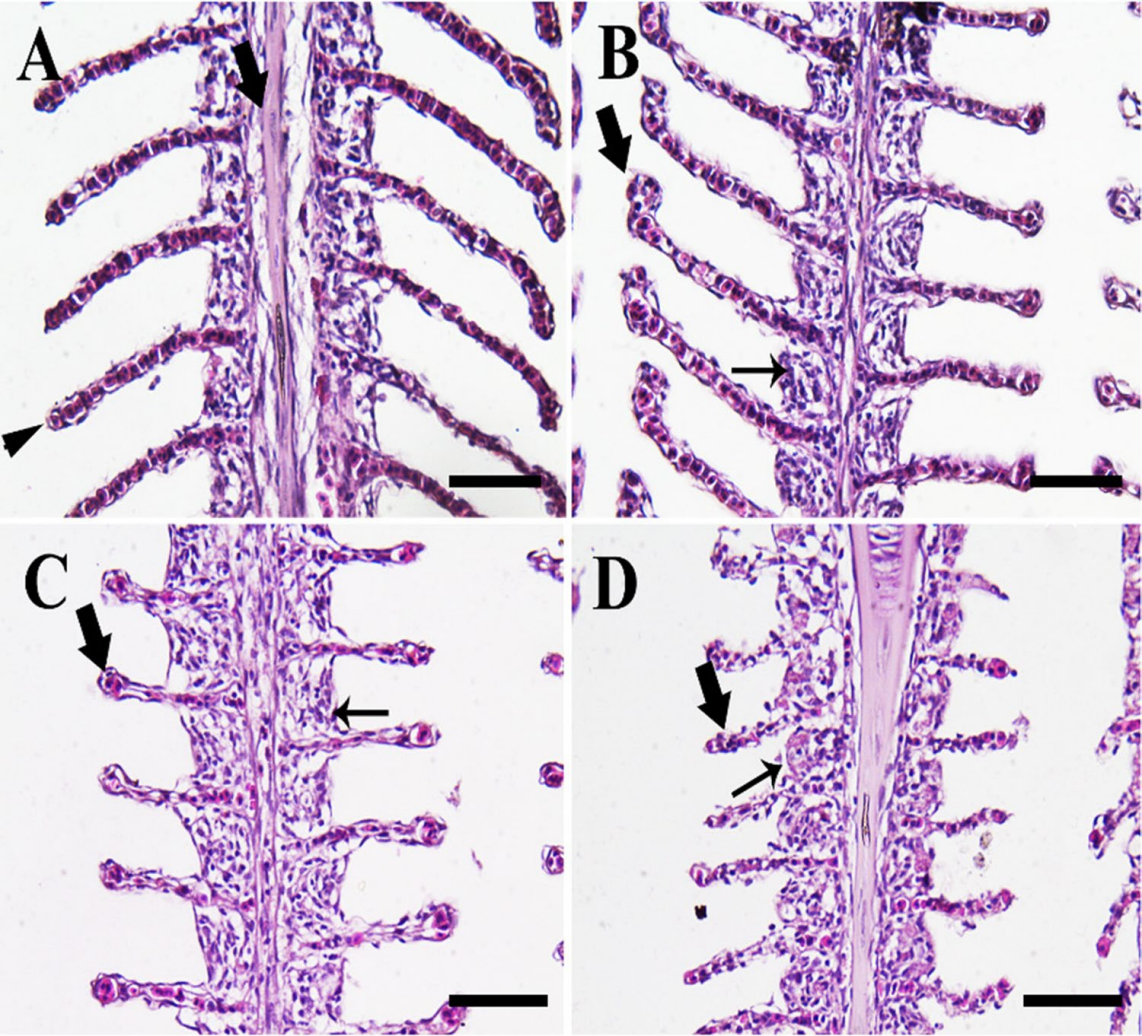
Histopathological examination of fish gills. **A** The 0-ppt group showing normal primary lamellae (arrow) and secondary lamellae (arrowhead). **B** Salinity (4 ppt) group revealing telangiectasis of secondary lamellae (thick arrow) and hypertrophy of chloride cells (thin arrow). **C** Salinity (8 ppt) group showing sever telangiectasis and necrosis of the secondary lamellae (thick arrow) and hypertrophy of chloride cells (thin arrow). **D** Salinity (12 ppt) group showing extensive necrosis of the secondary lamellae (thick arrow) and hypertrophy of chloride cells (thin arrow). *Scale bar* = 50 μm

Fish reared in the 0-ppt group showed the normal gill architecture with normal primary and secondary lamellae (Fig. 2A). On the other hand, fish in the 4-ppt group revealed telangiectasis of the secondary lamella and hypertrophy of chloride cells (Fig. 2B). Besides, fish in the 8-ppt group showed excessive telangiectasis, necrosis of the secondary lamellae, and hypertrophy of chloride cells (Fig. 2C). Furthermore, fish in the 12-ppt group were exposed to severe hypertrophy of chloride cells with severe necrosis of the secondary lamellae (Fig. 2D).

**Fig. 3:**
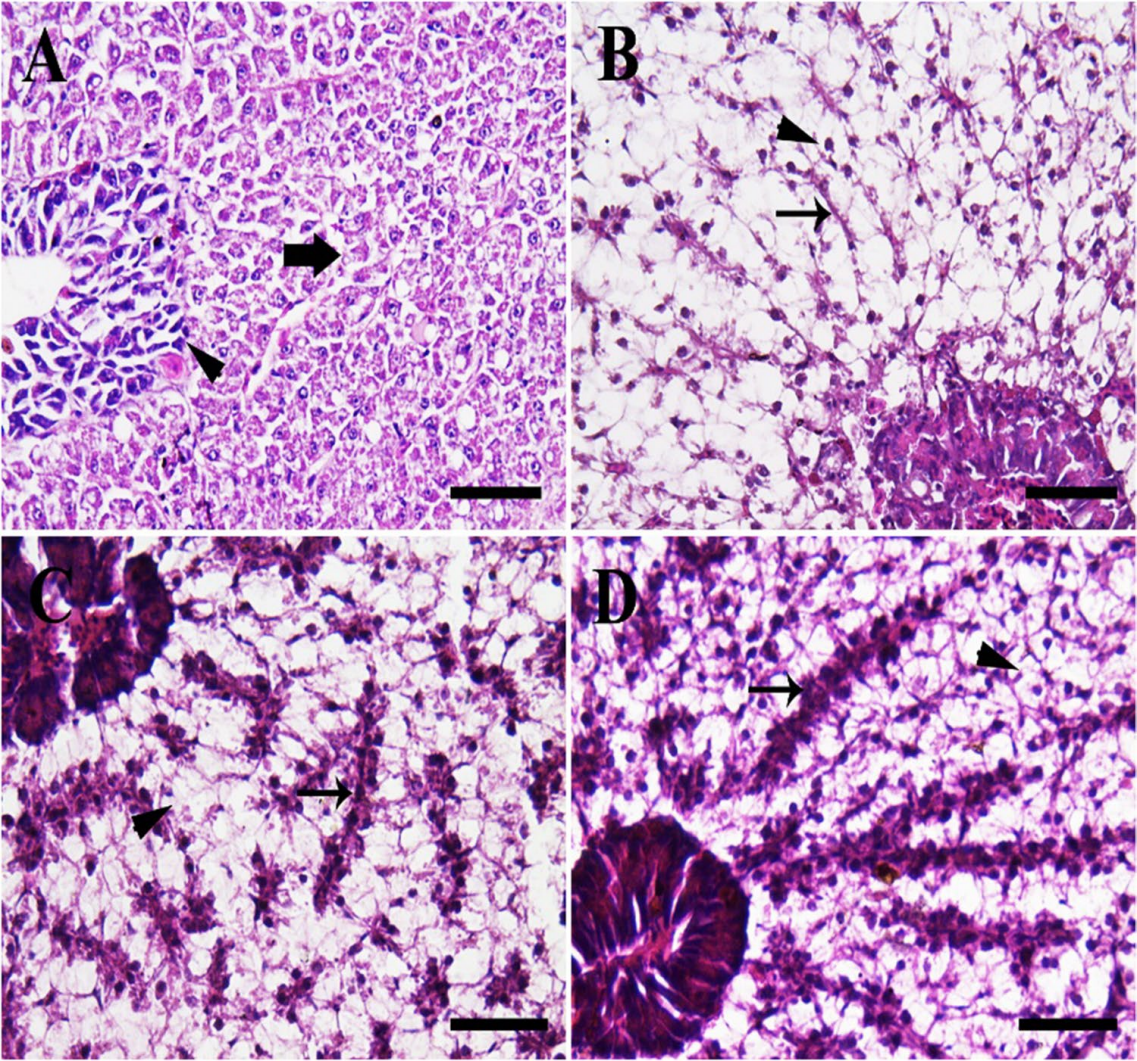
Histopathological examination of fish liver. **A** The 0-ppt group revealing normal hepatocytes (thick arrow) and normal pancreatic acini (arrowhead). **B** Salinity (4 ppt) group exposing slight vascular congestion (thin arrow) and fatty vacuolized hepatocytes with pyknotic nuclei (arrowhead). **C** Salinity (8 ppt) group revealing moderate congestion of hepatic sinusoid (thin arrow) and moderate number of necrotic hepatocytes (arrowhead). **D** Salinity (12 ppt) group showing extensive congestion of hepatic sinusoid (thin arrow) and high number of pyknotic hepatic nuclei (arrowhead). *Scale bar* = 50 μm

Fish reared in the 0-ppt group showed the normal hepatopancreatic architecture with normal hepatic cord and acini of the exocrine pancreas (Fig. 3A). However, fish in the 4-ppt group revealed slight vascular congestion and diffuse fatty vacuolized hepatocytes with pyknotic nuclei (Fig. 3B). In addition, fish in the 8-ppt group showed a moderate number of necrotic nuclei of hepatocytes and moderate congestion of hepatic sinusoid (Fig. 3C). Moreover, fish in the 12-ppt group revealed severe hepatic sinusoid congestion with diffuse fatty vacuolized necrotic hepatocytes (Fig. 3D).

### Antioxidative capacity (SOD, CAT, and GSH) and lipid peroxidation marker (MDA)

**Fig. 4:**
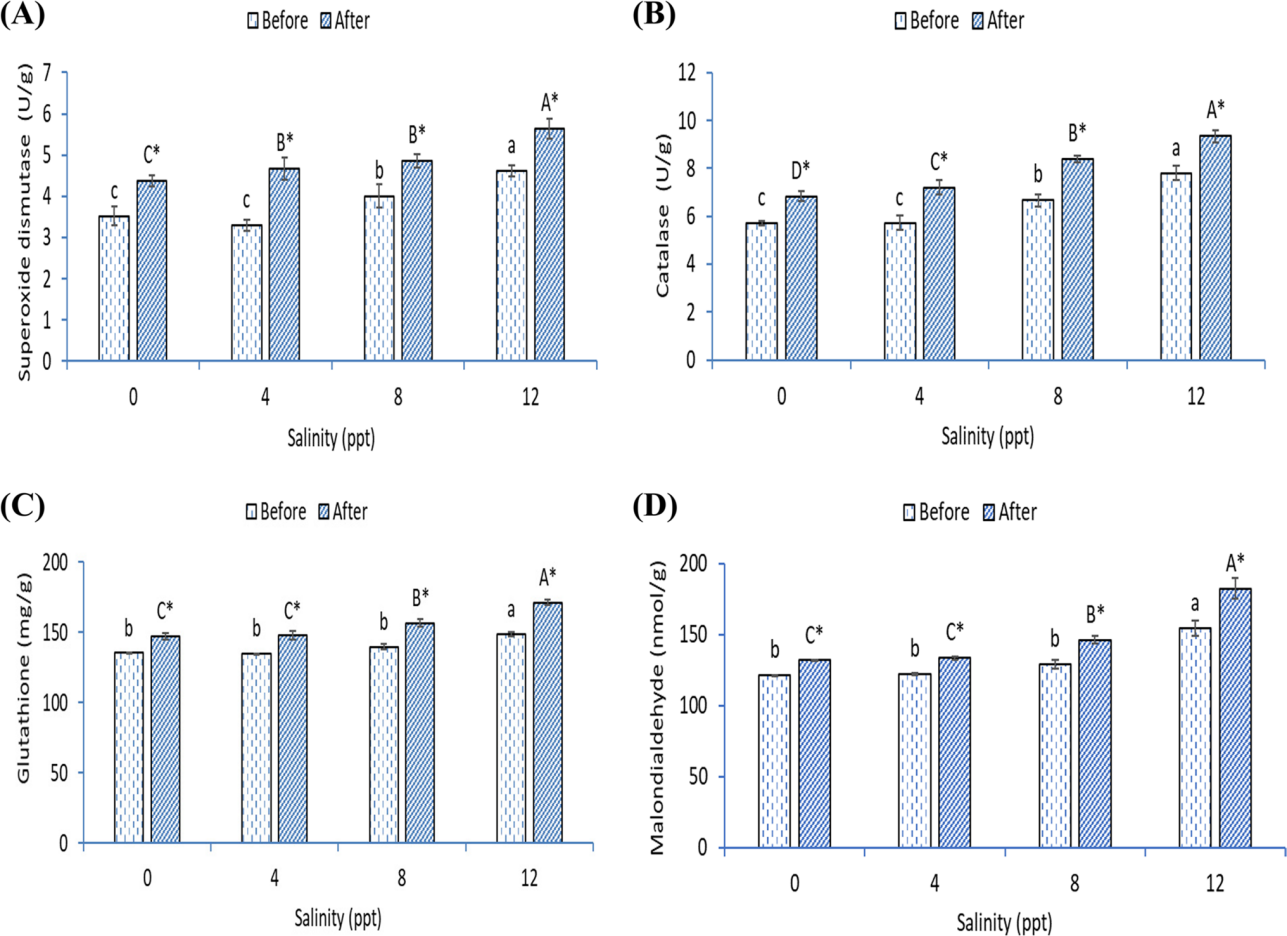
Intestinal (**A**) superoxide dismutase, (**B**) catalase, (**C**) glutathione activities, and (**D**) malondialdehyde level of African catfish exposed with varying levels of salinity and heat stress. Bars with different small or capital letters differ significantly either before or after the heat stress (*p* < 0.05). The asterisk (*) refers to significant differences between the same groups before and after heat stress (*p* < 0.05)

The intestinal superoxide dismutase (SOD) (Fig. 4A), catalase (CAT) (Fig. 4B), glutathione (GSH) (Fig. 4C), and malondialdehyde (MDA) (Fig. 4D) were markedly increased in African catfish stressed with 12-ppt salinity (*p* < 0.05). Before heat stress, the activities of SOD and CAT were higher in fish exposed to 8 ppt than fish in 0- and 4-ppt groups and lower than fish in 12 ppt (*p* < 0.05). Also, fish exposed to 12 ppt had higher GSH and MDA than fish grown in 0, 4, and 8 ppt. After heat stress, in all groups (0, 4, 8, and 12 ppt), SOD, CAT, GSH, and MDA were markedly increased compared with before heat stress (*p* < 0.05). The activity of SOD was higher in fish exposed to 4 and 8 ppt than fish in the 0-ppt group and lower than fish in 12 ppt (*p* < 0.05). Further, CAT was increased markedly and gradually by increasing the salinity level (*p* < 0.05). The activities of GSH and MDA were higher in fish exposed to 8 ppt than fish in 0- and 4-ppt groups and lower than fish in 12 ppt (*p* < 0.05). Before or after heat stress, fish exposed with 12-ppt salinity showed the highest SOD, CAT, GSH, and MDA before or after heat stress (*p* < 0.05).

**Fig. 5:**
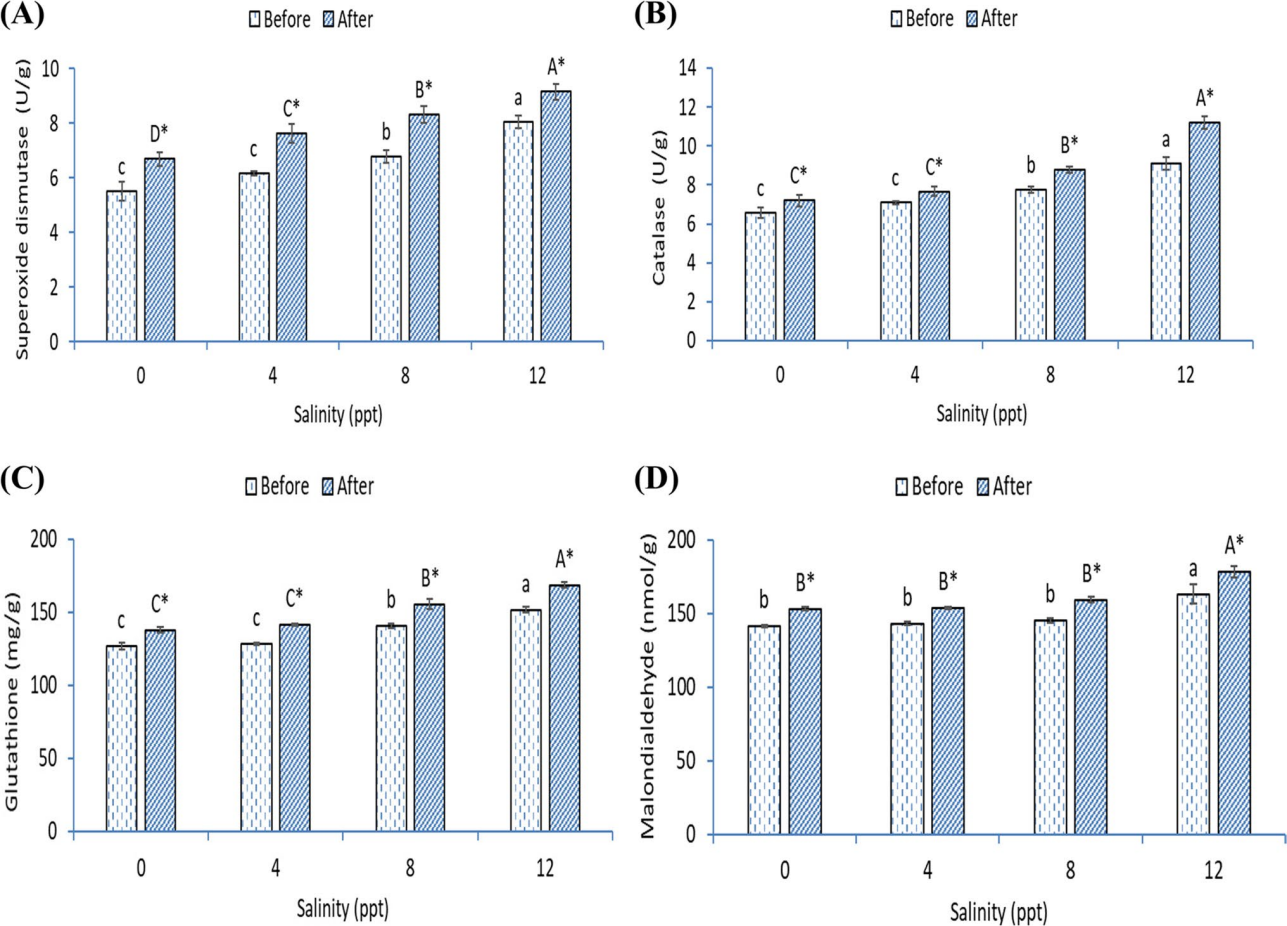
Gill (**A**) superoxide dismutase, (**B**) catalase, (**C**) glutathione activities, and (**D**) malondialdehyde level of African catfish exposed with varying levels of salinity and heat stress. Bars with different small or capital letters differ significantly either before or after the heat stress (*p* < 0.05). The asterisk (*) refers to significant differences between the same groups before and after heat stress (*p* < 0.05)

The samples of gill homogenates showed higher SOD (Fig. 5A), CAT (Fig. 5B), and GSH (Fig. 5C) in fish exposed with 8- and 12-ppt salinity than fish in 0- and 4-ppt groups before heat stress (*p* < 0.05). Further, the levels of MDA (Fig. 5D) were meaningfully higher in the 12-ppt group than the 0-, 4-, and 8-ppt groups (*p* < 0.05). After heat stress, all fish groups showed higher SOD, CAT, GSH, and MDA values than before heat stress (*p* < 0.05). Further, SOD was increased markedly and gradually by increasing the salinity level (*p* < 0.05). The activities of CAT and GSH were higher in fish exposed to 8 ppt than fish in 0- and 4-ppt groups and lower than fish in 12 ppt (*p* < 0.05). Fish exposed with 12-ppt salinity showed the highest MDA level after heat stress (*p* < 0.05).

**Fig. 6:**
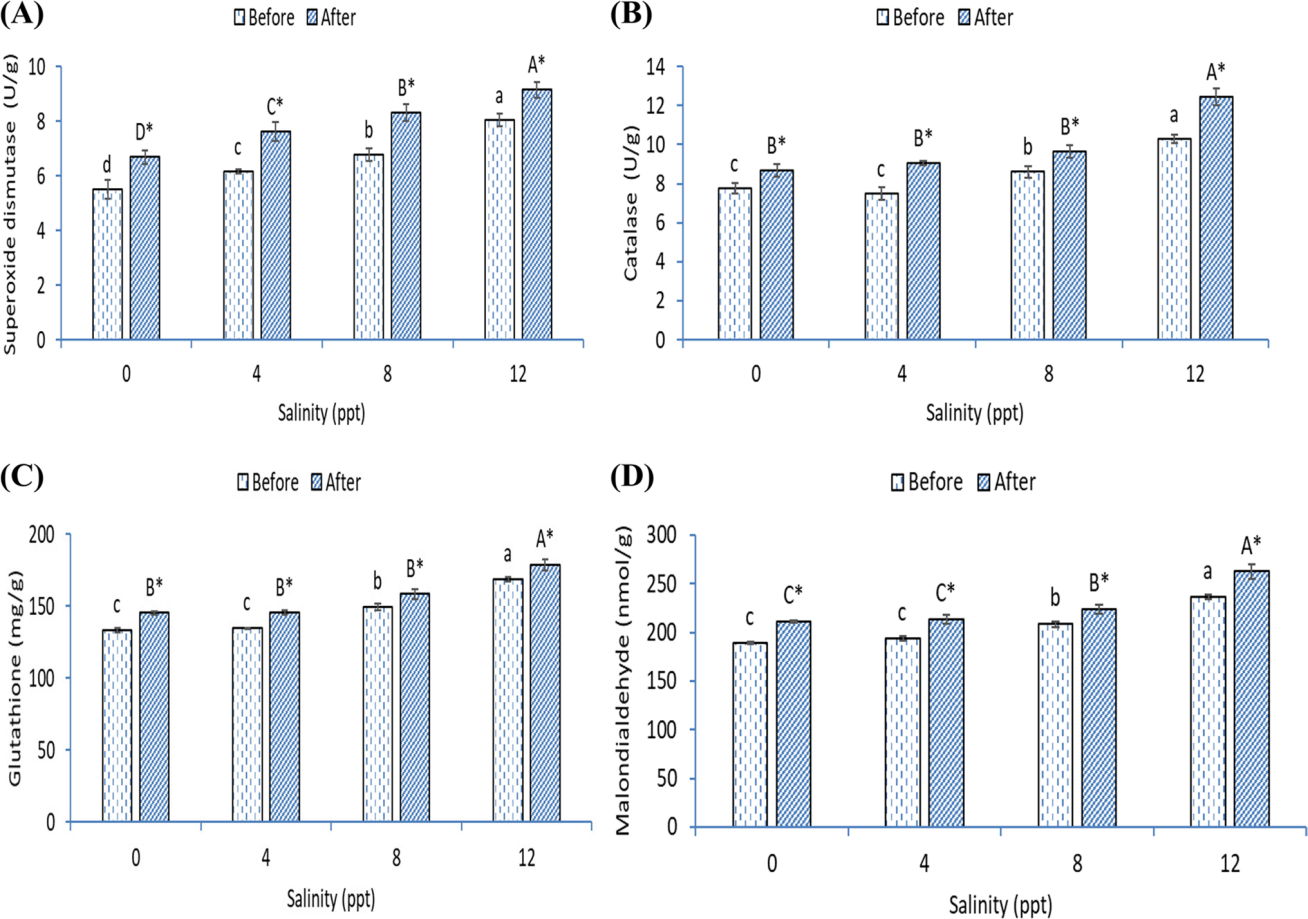
Liver (**A**) superoxide dismutase, (**B**) catalase, (**C**) glutathione activities, and (**D**) malondialdehyde level of African catfish exposed with varying levels of salinity and heat stress. Bars with different small or capital letters differ significantly either before or after the heat stress (*p* < 0.05). The asterisk (*) refers to significant differences between the same groups before and after heat stress (*p* < 0.05)

The activity of SOD (Fig. 6A) was increased markedly and gradually by increasing the salinity level before and after heat stress (*p* < 0.05). Before heat stress, liver CAT (Fig. 6B), GSH (Fig. 6C), and MDA (Fig. 6D) have increased in fish of 8- and 12-ppt groups than fish in 0- and 4-ppt groups and lower than fish in 12 ppt (*p* < 0.05). Fish in the 8-ppt group had lower CAT, GSH, and MDA than fish in the 12-ppt group (*p* < 0.05). After heat stress, all groups showed a marked increased SOD, CAT, GSH, and MDA than fish before heat stress in the same manner (*p* < 0.05). After heat stress, fish exposed with 12-ppt salinity showed the highest CAT and GSH activities (*p* < 0.05). MDA levels were higher in fish exposed to 8 ppt than fish in 0- and 4-ppt groups and lower than fish in 12 ppt (*p* < 0.05).

### Blood biochemistry variables

**Fig. 7:**
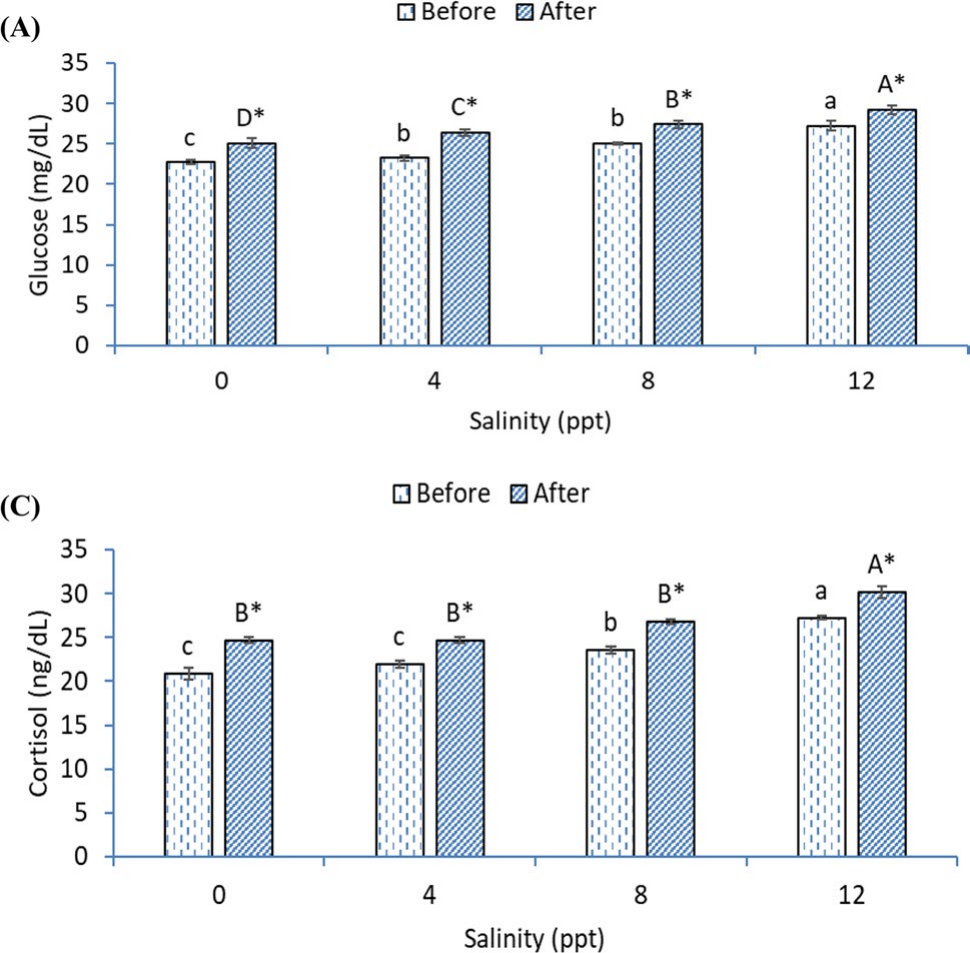
Blood glucose (**A**) and cortisol (**B**) levels of African catfish exposed with varying levels of salinity and heat stress. Bars with different small or capital letters differ significantly either before or after the heat stress (*p* < 0.05). The asterisk (*) refers to significant differences between the same groups before and after heat stress (*p* < 0.05)

The levels of blood glucose were markedly increased in fish exposed with 4, 8, and 12 ppt than 0 ppt in a gradual manner either before or after heat stress (*p* < 0.05; Fig. 7A). The cortisol level was markedly increased in 8- and 12-ppt groups before heat stress while increasing only 12 ppt after heat stress (*p* < 0.05; Fig. 7B). The glucose and cortisol levels were markedly increased in all groups after heat stress compared with before heat stress.

The values of ALT and urea were increased in the blood samples of African catfish in 8 and 12 ppt before and after heat stress (*p* < 0.05; Table 2). At the same time, AST activity and creatinine levels were increased in the 12-ppt group before heat stress. After heat stress, AST was increased in 8- and 12-ppt groups while creatinine increased in the 12-ppt group (*p* < 0.05; Table 2). Blood total protein was increased in 8- and 12-ppt groups before heat stress, but no differences were seen among the groups after heat stress (*p* < 0.05; Table 2). The albumin level was increased in the 12-ppt group before and after heat stress (*p* < 0.05; Table 2). The globulin levels were higher in 8- and 12-ppt groups than 0- and 4-ppt groups before and after heat stress (*p* < 0.05; Table 2). After heat stress, all groups showed marked differences for all blood biochemical traits compared with before heat stress. Significant salinity and heat stress interactions were seen on the ALT, AST, urea, creatinine, total protein, albumin, and globulin values (*p* < 0.05).

**Table 2:** Blood biochemical variables of African catfish exposed with varying levels of salinity and heat stress

	ALT (U/I)	AST (U/I)	Total protein (g/dL)	Albumin (g/dL)	Globulin (g/dL)	Urea (mg/dL)	Creatinine (mg/dL)
Before heat stress							
0 ppt	21.98 ± 0.71 c	24.13 ± 0.58 b	4.09 ± 0.02 a	2.18 ± 0.04 a	1.91 ± 0.06 a	2.19 ± 0.04 c	0.32 ± 0.01 b
4 ppt	21.46 ± 0.56 c	23.96 ± 0.30 b	4.07 ± 0.03 a	2.24 ± 0.04 a	1.83 ± 0.06 a	2.21 ± 0.05 c	0.32 ± 0.01 b
8 ppt	24.36 ± 0.65 b	24.94 ± 0.93 b	3.79 ± 0.07 b	2.11 ± 0.02 a	1.68 ± 0.09 b	2.41 ± 0.02 b	0.37 ± 0.01 b
12 ppt	27.25 ± 0.50 a	27.67 ± 0.49 a	3.56 ± 0.07 b	1.92 ± 0.07 b	1.64 ± 0.14 c	2.60 ± 0.08 a	0.41 ± 0.01 a
After heat stress							
0 ppt	25.33 ± 0.61 C*	25.96 ± 0.47 C*	3.70 ± 0.04*	2.05 ± 0.04 A*	1.65 ± 0.06 A*	2.30 ± 0.04 C*	0.40 ± 0.01 B*
4 ppt	24.77 ± 0.20 C*	25.75 ± 0.19 C*	3.72 ± 0.03*	2.05 ± 0.01 A*	1.68 ± 0.02 A*	2.41 ± 0.03 C*	0.41 ± 0.01 B*
8 ppt	26.77 ± 0.43 B*	27.14 ± 0.94 B*	3.52 ± 0.05*	1.93 ± 0.04 AB*	1.59 ± 0.08 B*	2.51 ± 0.03 B*	0.43 ± 0.01 B*
12 ppt	29.23 ± 0.52 A*	29.39 ± 0.46 A*	3.33 ± 0.01*	1.81 ± 0.02 B*	1.52 ± 0.02 B*	2.74 ± 0.09 A*	0.48 ± 0.01 A*
Two-way ANOVA (*p*-value)							
Salinity	0.001	0.001	0.001	0.001	0.001	0.001	0.001
Heat stress	0.001	0.001	0.001	0.001	0.001	0.001	0.001
Interaction	0.001	0.001	0.001	0.001	0.001	0.001	0.001

Means ± S.E. in the same column with different small or capital letters differ significantly either before or after the heat stress (*p* < 0.05). The asterisk (*) refers to significant differences between the same groups before and after heat stress (*p* < 0.05). *AST* aspartate aminotransferase *ALT* alanine aminotransferase

### Integrated biomarker response

**Fig. 8:**
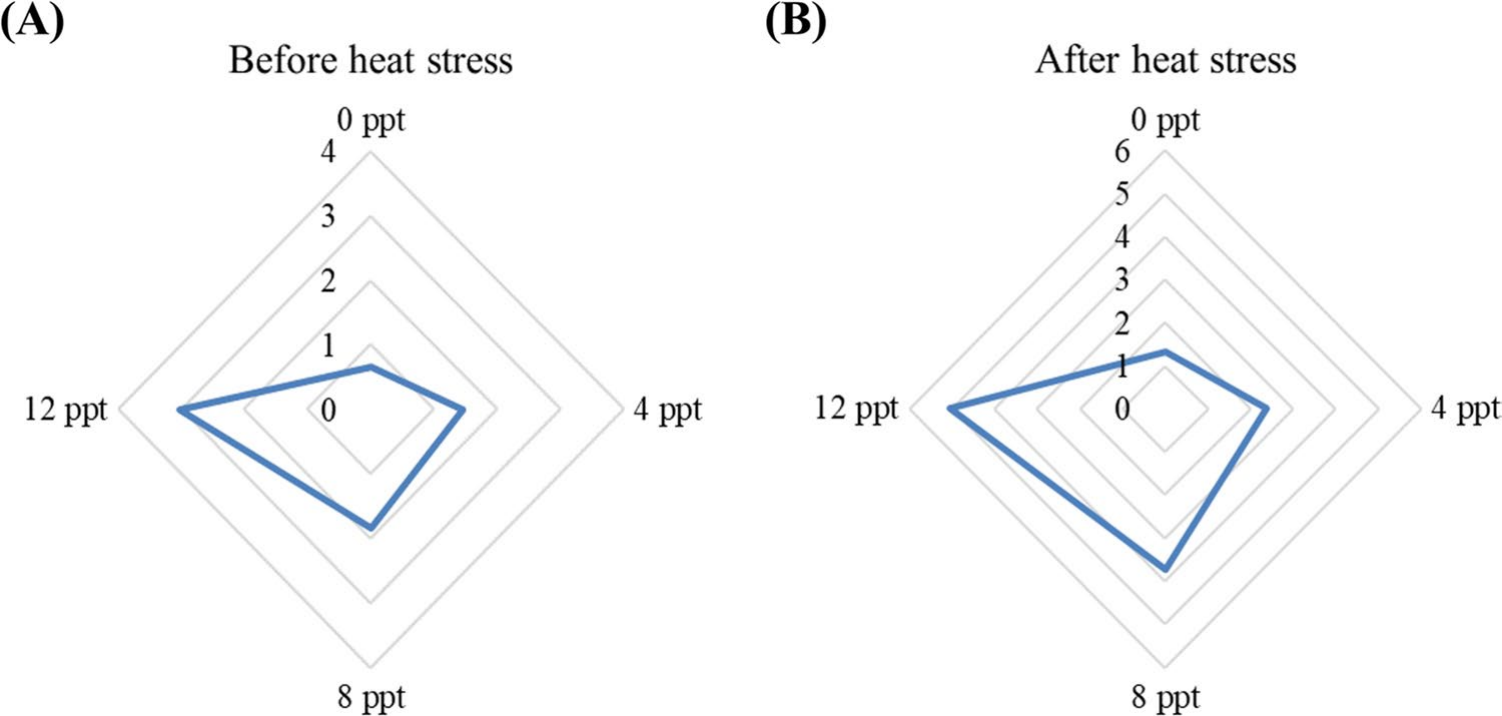
Integrated biomarker response (IBR) of African catfish exposed with varying levels of salinity and before and after heat stress

The integrated multi-biomarker response (IBR) results are shown in Table 3 and Fig. 8. The results showed marked differences among the groups gradually before and after heat stress. Before and after heat stress, the highest IBR was seen in African catfish exposed with 12 ppt, while the lowest IBR was in the 0-ppt group (*p* < 0.05).

**Table 3:** Integrated biomarker response (IBR) of African catfish exposed with varying levels of salinity and heat stress

	Median	Mean	SD	Min	Max
Before heat stress					
0 ppt	0.66 d	0.66	0.11	0.40	0.92
4 ppt	1.47 c	1.47	0.23	1.22	1.71
8 ppt	1.85 b	1.85	0.19	1.58	2.11
12 ppt	3.03 a	3.03	0.21	2.16	3.89
After heat stress					
0 ppt	1.34 D*	1.34	0.11	1.02	1.65
4 ppt	2.37 C*	2.37	0.23	2.05	2.68
8 ppt	3.74 B*	3.74	0.19	3.21	4.26
12 ppt	5.06 A*	5.06	0.21	3.89	6.22
Two-way ANOVA (*p*-value)					
Salinity	0.001	-	-	-	-
Heat stress	0.001	-	-	-	-
Interaction	0.001	-	-	-	-

Means ± S.E. in the same column with different small or capital letters differ significantly either before or after the heat stress (*p* < 0.05). The asterisk (*) refers to significant differences between the same groups before and after heat stress (*p* < 0.05)

## Discussion

Aquaculture activity is not far from the fluctuations in the environmental changes associated with influences on the water quality and its relationship with fish health (Reid et al., 2019a). Usually, fish suffer from several stressors in the farms, such as fluctuations in the water salinity, ammonia accumulations, and dissolved oxygen (Deane and Woo, 2009; Shukry et al., 2021). Accordingly, it is mandatory to investigate the impacts of unstable environmental conditions on finfish species’ performance to sustain fish production (Ahmed et al., 2019; Reid et al., 2019b). Growing freshwater fish species is not available in some areas due to less availability of water resources. Alternatively, brackish water can grow fish, but this depends on fish species and the stability of other environmental conditions (e.g., temperature, ammonia, and stocking density) (Mitra, 2013). African catfish is a popular commercial fish species with a high capacity to adapt to diverse environmental conditions (Dauda et al., 2018). However, high salinity and heat stress are proposed to impair fish performances and health status, leading to low productivity and well-being (Eissa and Wang, 2016). In this study, African catfish were grown in varying water salinities (0, 4, 8, and 12 ppt) for 4 weeks then exposed to heat stress (32 °C). The results showed the marked impact of high salinity on the growth performance and interactive influences of water salinity and heat stress on the health condition of African catfish. Up to 8-ppt fish showed no significant differences with fish grow in 0 and 8 ppt in the final body weight, specific growth rate, FCR, and survival rate. However, fish reared in 12 ppt had impaired growth performance, FCR, and survival rate. The results agree with various studies that indicated that catfish requires optimal water salinity for normal growth. Trong et al. (2017) reported that catfish (*Pangasianodon hypophthalmus*) reared in high salinity (12 ppt) had impaired growth performance. The authors attributed the reduced growth performance to the osmoregulatory budget requirements, which need high energy to adapt to stressful conditions (Dawood et al., 2021b; Mohamed et al., 2021). Fish require high energy under hypoosmotic or hyperosmotic environments that can affect the metabolic and growth promotion activity, leading to less growth performance and a high mortality rate (Abass et al., 2016). The reduced growth performance is also attributed to high salinity in disturbing the osmoregulation in the intestines of fish, leading to less feed utilization (Islam et al., 2020). Concurrently, the results showed high FCR in the groups of fish reared in high salinity compared to the remaining groups. The reduced survival rate in this study is a feature of low feed utilization and impaired health status.

Blood biochemical indices, antioxidant markers, and histological features are reliable and indicative indices correlated with the impact of stressors on fish physiological and productive status (Šimková et al., 2015). The impact of water salinities with or without heat stress on the health status of African catfish was evaluated by detecting biochemical blood indices, oxidative-related markers, and histological features in the intestines, gills, and livers. The primary role of gills and intestines is the osmoregulation and hyposalinity, or hypersalinity led to disturbed osmoregulation capacity, thereby disturbances in fish’s metabolic and physiological function (Ern and Esbaugh, 2018; Rivera-Ingraham and Lignot, 2017; Webb and Wood, 2000). In this study, intestine, gill, and liver tissues showed impaired histological features attributed to the impact of high salinity (12 ppt) on the health status of African catfish. The abnormalities in the intestine, gill, and liver tissues of African catfish can be explained by salinity-induced oxidative stress (Dawood et al., 2021b). Stressful conditions cause the generation of free radicals, peroxides, and reactive oxygen metabolites (ROS) involved in lipid peroxidation, DNA damage, and cell mortality (Blewett et al., 2016; Chang et al., 2021b). The stressful conditions induce high secretion of cortisol which helps release glucose as a source of energy (Bonga, 1997). High lipid peroxidation is expressed by high malondialdehyde secretion (MDA). In this case, cells develop enzymatic and non-enzymatic activities to degenerate the excessive free radicals and ROS (Kim et al., 2017). Superoxide dismutase (SOD), catalase (CAT), and glutathione (GSH) are among the main biomarkers responsible for relieving the impact of oxidative stress on the organism’s entire body (Wang et al., 2016). The current study showed that the antioxidants (SOD, CAT, and GSH) increased with an increase in MDA levels. Although the synthesis of antioxidative molecules is increased, it is insufficient to prevent tissue peroxidation (MDA) and simultaneous change in tissue architecture, as reflected from the histological study of three tissues. The increased MDA level in this study explains the abnormalities in the intestine, gill, and liver organs (Mohamed et al., 2021). Additionally, in this study, cortisol and glucose levels were markedly increased in African catfish reared in high salinity with or without heat stress. The results are concurrent with Trong et al. (2017), who stated that catfish (*P. hypophthalmus*) grown in hypersalinity and the high temperature had high glucose and cortisol levels.

When disturbances occur in the liver tissue, the release of its metabolites and enzymes is also disrupted (Chang et al., 2021a). In this study, blood ALT and AST activities were higher in fish in hypersalinity with or without heat stress. High ALT and AST levels indicated the liver dysfunction that the effect of oxidative stress might induce (Ghelichpour et al., 2020). Similarly, the renal tissue-related indices (urea and creatinine) were higher in fish stressed with hypersalinity with or without heat stress (Abdel-Latif et al., 2021; Waheed et al., 2020). The high creatinine levels are related to the breaking of creatinine in the fish’s muscles then go through the kidney out the fish body (Patel et al., 2013). At the same time, urea indicates the excessive rate of broken tissues and the high metabolic rate in stressed fish bodies (Hazon et al., 2003; Wilkie, 2002).

The integrated biomarker response (IBR) is suitable for assessing the impact of various stressors on fish’s physiological and health status (Perussolo et al., 2019). IBR can present the response of fish to stress in only one value that can help understand the overall impact of stress on fish performances. The high value of IBR refers to the high impact of stress on the physiological condition of fish. In parallel, the IBR in African catfish stressed with high salinity with or without heat stress increases with increasing water salinity. The results agree with Dawood et al. (2021b), who indicated that the IBR value increased in *Nile tilapia* stressed with high salinity and exposed with hypoxia stress.

## Conclusion

In summary, growing African catfish in high salinity (12 ppt) hampered the growth performance and health status. The histological evaluation of the intestines, gills, and livers of African catfish showed normal features in fish grow in 0, 4, and 8 ppt but severe alterations in fish raised in 12 ppt. After salinity and heat stress, African catfish reared in high salinity (12 ppt) responded with higher production of both antioxidative molecules but not to the level that could check the lipid peroxidation and simultaneous tissue histopathological stress-related markers. Further, liver and kidney-related markers were high in fish stressed with high salinity and heat stress. The obtained results indicate the necessity of optimizing water salinity and temperature for the optimum growth performance and well-being of African catfish.
